# Recovery of Antioxidants from Tomato Seed Industrial Wastes by Microwave-Assisted and Ultrasound-Assisted Extraction

**DOI:** 10.3390/foods11193068

**Published:** 2022-10-03

**Authors:** Ignacio Solaberrieta, Cristina Mellinas, Alfonso Jiménez, María Carmen Garrigós

**Affiliations:** Department of Analytical Chemistry, Nutrition & Food Sciences, University of Alicante, San Vicente del Raspeig, ES-03690 Alicante, Spain

**Keywords:** tomato seed wastes, antioxidant compounds, microwave-assisted extraction, ultrasound-assisted extraction, response surface methodology, Box–Behnken design

## Abstract

Tomato seed (TS) wastes are obtained in large amounts from the tomato processing industry. In this work, microwave-assisted extraction (MAE) and ultrasound-assisted extraction (UAE) of antioxidant compounds from TS were optimized by using response surface methodology. The effect of MAE and UAE main extraction parameters was studied on total phenolic content (TPC) and antioxidant activity (DPPH) responses. Antioxidant, structural, morphological, and thermal properties of MAE and UAE extracts were evaluated. A great influence of ethanol concentration was observed in both extraction methods. Optimal MAE conditions were determined as 15 min, 80 °C, 63% ethanol and 80 mL, with a desirability value of 0.914, whereas 15 min, 61% ethanol and 85% amplitude (desirability = 0.952) were found as optimal conditions for UAE. MAE extracts exhibited higher TPC and antioxidant activity values compared to UAE (1.72 ± 0.04 and 1.61 ± 0.03 mg GAE g TS^−1^ for MAE and UAE, respectively). Thermogravimetric analysis (TGA) results suggested the presence of some high molecular weight compounds in UAE extracts. Chlorogenic acid, rutin and naringenin were identified and quantified by HPLC-DAD-MS as the main polyphenols found by MAE and UAE, showing MAE extracts higher individual phenolics content (1.11–2.99 mg 100 g TS^−1^). MAE and UAE have shown as effective green techniques for extracting bioactive molecules with high antioxidant activity from TS with high potential to be scaled-up for valorizing of TS industrial wastes.

## 1. Introduction

The use of active biomolecules obtained from agricultural by-products or wastes, as renewable sources for the development of innovative added-value products, has gained great importance due to its environmental and economic advantages [1,2,3,4]. According to Hills et al. [5], 140 Gt of agricultural residues is annually generated worldwide, being considered an unavoidable source of potential resources [6]. Moreover, the FAO projections to 2030 include a continuous expansion of the agricultural sector in developing countries, where there exists a positive correlation between crop residue availability and production. Several agro-wastes, such as tomato peels and seeds [7,8,9,10], almond skins [11], cocoa bean shells [12], carob pods [13], coffee grounds [14], pomegranate [15] and pomelo peels [16], among many others, have been reported to contain significant amount of biomolecules which, once extracted from the vegetal matrices, could be potentially applied for the development of innovative materials for different applications, such as the following: food packaging or edible coatings; functional food additives or flavorings [17,18,19,20]; nutraceuticals and cosmetics [3].

Among these crops, tomato stands out as one of the most widely consumed vegetables in the world, either in raw form or in processed products. During tomato processing, a considerable amount of waste is generated, which is mainly composed of peels and seeds. These by-products are usually disposed of in landfills and only partially reused by composting or for animal feeding [7]. However, these wastes represent a sustainable source for the extraction of added value chemicals, such as polyphenols [21,22,23,24], fatty acids [25,26], proteins [27,28], pectin [29,30], carotenoids [10,31], tocopherols [21,32], and cutin [33]. Therefore, tomato wastes and by-products have huge potential for obtaining high-value functional ingredients, and the sequential extraction approach for these compounds constitutes a promising way to fully valorize this under-utilized biomass.

Conventional extraction techniques have been widely used to obtain several biomolecules from vegetal matrices. However, they present major drawbacks such as being time consuming and using large volumes of organic solvents [34,35,36]. In recent years, alternative and more environmentally friendly extraction techniques have been developed. Among them, microwave-assisted extraction (MAE) and ultrasound-assisted extraction (UAE) have gained major importance due to their multiple advantages compared to conventional extraction techniques, leading to increased extraction yields and reduced extraction time and solvent consumption.

Moreover, MAE and UAE are both scalable, and they have been extensively used for the extraction of several biomolecules from different natural sources, including tomato by-products and wastes [8,37,38,39]. However, these studies have mainly focused on tomato peel wastes or a peels and seeds mixture (tomato pomace). Recently, Szabo et al. [40] studied the influence of cold break and hot break thermal treatments on tomato processing to obtain bioactive compounds present in tomato peels and oil seeds, reporting an important lycopene degradation in tomato peels as well as a decrease in carotenoids content in the oil samples by using high temperatures (85–95 °C). Tomato peel has been widely investigated as a source of polyphenols, pectin, and fatty acids [7,8,41,42], suggesting a great potential for the valorization of this waste for different applications, such as food packaging [43] or medicine [44]. MAE and UAE have been previously used to obtain polyphenolic compounds from tomato peels. For example, Bakić et al. [8] studied the effect of different solvents in MAE, obtaining better results by using 70% (*v*/*v*) methanol with 1% (*v*/*v*) HCl at 90 °C in terms of total phenols, total flavonoids, and individual phenolic compounds yields. In another work, UAE was applied by Grassino et al. to sequentially obtain pectin and polyphenols from tomato peels, showing that polyphenols extraction was significantly influenced by solvent polarity, and obtaining better efficiency by using 70% ethanol compared to 96% ethanol [41].

To the best of our knowledge, a specific study on tomato seed wastes to obtain polyphenolic compounds with antioxidant activity using MAE and UAE techniques has not been reported up to now. So, the present research represents a significant contribution to the valorization of seed wastes derived from the tomato industry, which present high economic and environmental impacts. In this study, a Box–Behnken design (BBD) was applied to evaluate the effect of the main factors affecting MAE and UAE of antioxidant compounds from tomato seed wastes on total phenolic content (TPC) and 2,2-diphenyl-1-picrylhydrazyl (DPPH) responses. Response surface models and simultaneous optimization using the desirability function were used to find the optimum extraction conditions in each process. MAE and UAE extracts obtained under optimal conditions were fully characterized and compared, for the first time, in terms of extraction yield; TPC and antioxidant activity using three independent methods (DPPH, ferric-reducing antioxidant power, FRAP, and 2,2’-azino-bis(3-ethylbenzothiazoline-6-sulfonic acid, ABTS) assays; thermal (TGA), structural (FTIR) and morphological (SEM) properties; as well as phenolics profile by HPLC-DAD-MS.

## 2. Materials and Methods

### 2.1. Raw Material and Reagents

Tomato seed wastes were provided by Stazione Sperimentale per l’Industria delle Conserve Alimentari (SSICA, Parma, Italy). Samples were lyophilized and ground into a powder using a Telstar Lyoquest−55 PLUS (Terrassa, Barcelona, Spain) and a ZM 200 high-speed rotatory mill (Retsch, Hann, Germany), respectively. Particles passing through a 1.0 mm sieve, to ensure the homogeneity of the sample, were used without any further treatment for polyphenols extraction. All chemicals were of analytical grade, and they were purchased from Sigma–Aldrich (Madrid, Spain).

### 2.2. Microwave-Assisted Extraction (MAE)

MAE was performed using a Milestone flexiWAVE^TM^ microwave oven (Milestone srl, Bergamo, Italy) in the open vessel configuration. The tomato seed (TS) powder amount was fixed to 1.0 g and samples were stirred at 400 rpm during extraction, according to preliminary experiments, using 2.45 GHz. Ethanol:water mixtures were used since ethanol has been reported as an efficient and low-toxic solvent for the extraction of phenolic compounds from different plant materials [11,45,46]. For the optimization of the extraction process, different combinations of solvent composition (%EtOH), extraction temperature (T), extraction time (t) and solvent volume (V) were used according to Table 1. After MAE, the obtained tomato seed extracts (TSE) were cooled to room temperature and centrifuged at 5300 rpm for 10 min. The solid residue was washed twice with the extraction solvent and then discarded. Then, the supernatant was pooled with the washing solvent and stored overnight at −20 °C in order to remove possible interferences, such as polysaccharides or proteins, by precipitation. Subsequently, ethanol was evaporated under reduced pressure, and water was eliminated by freeze-drying in order to obtain a dried tomato seed extract. TSE solutions were freshly prepared, before analyses, at 2000 mg kg^−1^ in ethanol:water (60%, *v*/*v*).

### 2.3. Ultrasound-Assisted Extraction (UAE)

UAE was performed using a UP400St Ultrasonic processor (Hielscher, Oderstrasse, Germany) equipped with a standard 524d22D sonotrode probe with a tip diameter of 22 mm. TS amount and extraction solvent volume were fixed to 2.0 g and 100 mL, respectively, considering previous tests. The ultrasonic probe was immersed in a 150 mL beaker, containing sample and solvent, and an ice bath was used for refrigeration. For the optimization of the extraction process, different combinations of solvent composition (%EtOH), extraction time (t) and amplitude (A) were used according to Table 2. After UAE, dried TSE were obtained following the experimental procedure detailed in Section 2.2.

### 2.4. Experimental Designs

MAE and UAE of phenolic compounds from TS were performed under different experimental conditions in order to determine the optimum extraction conditions, which simultaneously maximize both antioxidant activity and total phenolic content. MAE optimization was performed by using a BBD with 29 runs and 5 central points to study the effect of four independent variables (% EtOH, T, t and V). Analogously, UAE optimization was carried out by using a BBD with 17 runs and 5 central points with three independent variables (%EtOH, t and A). In both cases, all experimental runs were performed randomly to minimize the effect of unexpected variability in the response variables. The range of the studied variables was selected on the basis of preliminary experiments, constructive characteristics of the used equipment and information in the literature [47,48,49,50]. Response surface methodology (RSM) was used, and regression analysis of experimental data was carried out by fitting an empirical second-order polynomial model to each response:

(1) $$Y=\beta_{0}+\sum \beta_{i}x_{i}+\sum \beta_{i}x_{i}^{2}+\sum \sum \beta_{ij}x_{i}x_{j}$$

where *Y* represents the predicted response variable, *X_i_* and *X_j_* represent the independent variables, *β*_0_ is a constant coefficient, and *β_i_*, *β_ii_*, *β_ij_* are the regression coefficients of linear, quadratic and interaction effect terms, respectively. Lack of fit test and coefficient of determination (R^2^) were used to determine the adequacy of the model to predict experimental data. Statistical significance of model parameters was determined at the 5% probability level (*α* = 0.05).

### 2.5. Scanning Electron Microscopy (SEM) Analysis

Morphology of dried TS powder was studied by SEM before and after MAE and UAE processes in order to evaluate the vegetal material damage produced during extraction experiments. Residues from MAE and UAE were oven-dried at 40 °C until constant mass and subsequently fixed on aluminum stubs. A SCD 004 Balzers sputter coater (Bal Tec., AG, Furstentum, Lichtenstein) was used to coat samples with a gold layer prior to analysis. SEM micrographs were obtained using a JEOL JSM 8400 scanning electron microscope (Peabody, MA, USA) at an accelerating voltage of 15 kV and 2500× magnification level.

### 2.6. Characterization of Tomato Seed Extracts (TSE)

#### 2.6.1. Extraction Yield

Extraction yield was gravimetrically determined by using the following equation:

Yield (%) = 100 m_TSE_/m_TS_ (2)

where m_TSE_ is the weight of extract obtained after freeze-drying, and m_TS_ is the weight of dried tomato seed powder.

#### 2.6.2. Total Phenolic Content (TPC)

Total phenolic content of TSE was determined using the Folin–Ciocalteu assay according to Toor and Savage [24], with some modifications. Aliquots (0.5 mL) of each extract were mixed with 2.5 mL of Folin–Ciocalteu reagent previously diluted in distilled water (1:10, *v*/*v*) and added with 2.0 mL of 7.5 wt% aqueous sodium carbonate. Then, the mixture was vortexed, and the absorbance was recorded at 765 nm after 30 min of incubation, at 45 °C, in the dark using a Biomate 3 UV-vis spectrophotometer (Thermospectronic, Mobile, AL, USA). Gallic acid in ethanol:water (60%, *v*/*v*) was used as reference standard for quantification (5–80 mg kg^−1^, R^2^ = 0.9991). Results were expressed as milligrams of gallic acid equivalents (GAE) per gram of TS. Each extract was analyzed in triplicate.

#### 2.6.3. Antioxidant Activity

DPPH scavenging activity of TSE was determined as described by Szabo et al. [50], with some modifications. Briefly, 0.4 mL of TSE was mixed with 2.1 mL of a freshly prepared DPPH solution (10^−4^ mol L^−1^ in ethanol). The mixture was vortexed and incubated, at room temperature, in the dark for 120 min. Then, the absorbance was measured at 517 nm against a pure ethanol blank. Trolox in ethanol:water (60%, *v*/*v*) was used as reference standard for quantification (5–70 mg kg^−1^, R^2^ = 0.9995). Results were expressed as milligrams of Trolox equivalents (TE) per gram of TS. Each extract was analyzed in triplicate.

ABTS assay was performed, in triplicate, according to Toor and Savage [24], with slight modifications. The ABTS radical cation was produced by mixing the ABTS solution (7 mM) with 2.45 mM potassium persulfate in a 1:1 ratio and allowing the mixture to stand in the dark, at room temperature, for 12 h. The ABTS working solution was obtained by diluting with aqueous ethanol (60%, *v*/*v*) to a final absorbance of 0.700 ± 0.001 at 734 nm. Then, 0.3 mL of TSE was mixed with 3 mL of the ABTS working solution, and the absorbance was measured after 120 min of incubation, at room temperature, in the dark. Trolox in EtOH:H_2_O (60%, *v*/*v*) was used as reference standard (5–60 mg kg^−1^, R^2^ = 0.9999). Results were expressed as milligrams of Trolox equivalents (TE) per gram of TS.

FRAP assay was determined according to Benzie and Strain [51]. The FRAP reagent was prepared by mixing 0.3 mol L^−1^ acetate buffer (pH = 3.6), 10 mmol L^−1^ TPTZ made up in 40 mmol L^−1^ HCl and 20 mmol L^−1^ FeCl_3_ at a 10:1:1 ratio. Then, 0.1 mL of TSE was mixed with 3 mL of the freshly prepared FRAP reagent pre-heated at 37 °C. The mixture was vortexed, and the absorbance was measured at 593 nm after 30 min of incubation at 37 °C. Trolox in EtOH:H_2_O (60%, *v*/*v*) was used as reference standard (5–100 mg/kg^−1^, R^2^ = 0.9999). Results were expressed as milligrams of Trolox equivalents (TE) per gram of TS. Each extract was analyzed in triplicate.

#### 2.6.4. Phenolic Profile by HPLC-DAD-MS

The identification and quantification of major phenolic compounds present in MAE and UAE optimized extracts were performed by high-performance liquid chromatography coupled to mass spectrometry (HPLC-DAD-MS). An Agilent 1100 HPLC system coupled to a LC/MSD ion trap mass spectrometer with electrospray ionization (ESI) source (Agilent Technologies, Palo Alto, CA, USA) was used. A HALO C_18_ column (100 mm × 4.6 mm × 2.7 µm) coupled to a HALO C_18_ guard column 90 Å (4.6 × 5 mm × 2.7 µm) operating at 25 °C was used to carry out the analysis at 294 nm. The mobile phase was composed of two solvents added with 0.1% (*v*/*v*) acetic acid (A: water and B: acetonitrile). The flow rate was 0.5 mL min^−1^ and the following gradient elution program was used: 0 min, 15% B; 0–15 min, 15–40% B; 15–18 min, 40–70% B; 18–19 min, 70–80% B; 19–20 min, 80% B; 20–22 min, 80–15% B (held 8 min). Mass spectra were recorded in the negative ionization mode (*m/z* 50–900). The electrospray chamber was set at 3.5 kV with a drying gas temperature of 350 °C. The N_2_ pressure and flow rate of the nebulizer were 50 psi and 10 L min^−1^, respectively. TSE and standard solutions were freshly prepared in EtOH:H_2_O (60%, *v*/*v*) and filtered through a 0.22 µm nylon membrane prior to injection. The injection volume was 6 µL and all analyses were carried out in triplicate. Extracted ion chromatograms and mass spectra experimental data were used for identification of polyphenols in TSE through comparison with standards. Quantitative analysis was performed using external calibration.

#### 2.6.5. Fourier-Transform Infrared Spectroscopy (FTIR)

FTIR spectra of optimized MAE and UAE extracts were recorded using a Bruker Analitik IFS 66/S spectrometer (Ettlingen, Germany) equipped with a KBr beam splitter and a DTGS detector. Spectra were collected in attenuated total reflectance (ATR) mode from 4000 to 400 cm^−1^ with an average of 64 scans at 4 cm^−1^ resolution.

#### 2.6.6. Thermal Properties

Thermogravimetric analysis (TGA) of MAE and UAE extracts was performed, in triplicate, with a TGA/SDTA 851 Mettler Toledo thermal analyzer (Schwarzenbach, Switzerland). Approximately 6 mg of sample was heated from 25 to 700 °C at 10 °C min^−1^ under nitrogen and oxygen atmospheres, both at 50 mL min^−1^ flow rate.

### 2.7. Statistical Analysis

All experiments were performed in triplicate and results are shown as mean values ± standard deviation (SD). Statgraphics Centurion XVI (Statistical Graphics, Rockville, MD, USA) was used to generate and analyze the BBD results. The graphic analysis of main effects and interactions between variables was used, and analysis of variance (ANOVA) was carried out. Differences between values were assessed based on confidence intervals by using the Tukey test at a *p* ≤ 0.05 significance level.

## 3. Results

### 3.1. Microwave-Assisted Extraction (MAE) Optimization

The experimental MAE conditions evaluated in the BBD and results obtained for the studied responses are shown in Table 1. Four independent variables were studied (ethanol concentration, solvent volume, extraction temperature and time) and the mathematical models obtained for TPC and DPPH responses by fitting experimental data and applying multiple regression analysis are presented in the following equations:

TPC (mg GAE g TS^−1^) = 1.01143 − 0.00407A + 0.01423B + 0.00589C − 0.00660D + 0.00015A^2^ − 0.00028AB + 0.00005AC + 0.00027AD − 0.00008B^2^ + 0.00001BC + 0.00007BD − 0.00012C^2^ + 0.00002CD + 3.7037^−7^D^2^ (3)

DPPH (mg TE g TS^−1^) = 0.64297 − 0.01260A − 0.00193B + 0.02044C − 0.00688D − 0.00007A^2^ − 0.00028AB + 0.00005AC + 0.00047AD − 0.00003B^2^ + 0.00011BC + 0.00008BD − 0.00022C^2^ + 0.00003CD − 0.00003D^2^ (4)

where A, B, C and D represent extraction time, temperature, ethanol concentration and solvent volume, respectively.

An analysis of variance (ANOVA) was performed to study the significance of experimental factors involved in the MAE of polyphenols from tomato seeds, as well as to evaluate the adequacy of the fitted models (Table 3) for TPC and DPPH responses (Equations (3) and (4), respectively). All the calculated mathematical models fitted adequately to the experimental data, since the lack of fit was not significant (*p* > 0.05), and acceptable R^2^ (0.9659 and 0.9044 for TPC and DPPH, respectively) and CV (4.89–7.25) values were obtained. Consequently, a high degree of correlation between experimental data and predicted values was observed, indicating that both models could be used to predict TPC and DPPH responses.

TPC values from TS by MAE ranged from 1.09 to 1.62 mg GAE g TS^−1^. The extraction temperature caused a significant positive effect (*p* < 0.001) on both TPC and antioxidant activity of TSE. High temperatures usually increase the solubility of active compounds, due to the acceleration of solvent diffusion into the sample matrix and, consequently, the extraction yield. Moreover, the cells of plant tissues may be destroyed at high temperatures accelerating solvent penetration through the plant material, allowing the release of active substances into the solvent which contribute to increase TPC and antioxidant activity [7,52,53,54,55].

Ethanol concentration exhibited significant positive and negative effects (*p* < 0.001) on DPPH and TPC responses, respectively. This different behavior could be related to the composition of the raw material, the dielectric constant of the solvent and the solubility of antioxidant compounds [56]. It has been suggested that the use of a binary solvent with lower organic fraction could be effective for polyphenols extraction. Madia et al. [57] reported that a low ethanol concentration allowed the co-extraction of other compounds, such as polysaccharides and proteins, facilitating the release of polyphenolic compounds. In addition, an increase in water content also increases the dielectric constant of the solvent, improving the degree of microwave absorption (by ionic conduction and dipole rotation mechanisms) compared to less polar solvents, resulting in higher polyphenols content [8]. In contrast, the obtained results for DPPH in this work suggested that not only polyphenols could be involved in the antioxidant activity of MAE extracts, as other non-phenolic compounds, contributing to the antioxidant activity, are able to be co-extracted at high ethanol contents [58]. It has been reported that a high concentration of ethanol may favor the extraction of antioxidant compounds present in tomato seeds with lower polarity such as carotenoids (lycopene) and vitamin E (α-tocopherol) [9,24,26,59]. Calvo et al. [60] also found that ethanol was more effective in extracting remaining lycopene in tomato peels by MAE compared to ethyl acetate.

**Figure 1:**
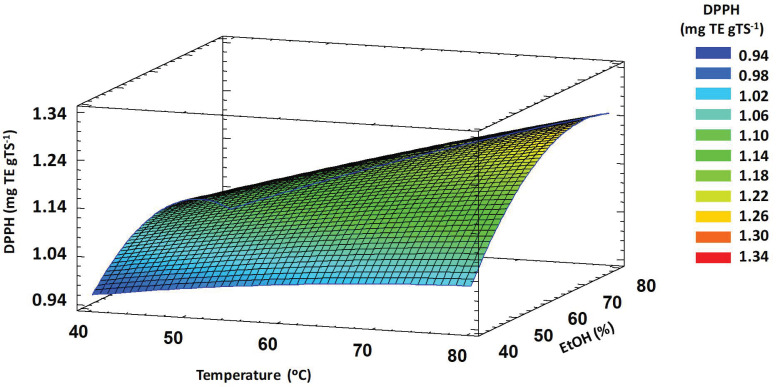
Response surface plot showing significant interaction of extraction temperature vs. ethanol concentration on DPPH antioxidant activity of TSE obtained by MAE. DPPH: 2,2-diphenyl-1-picrylhydrazyl, TSE: tomato seed extracts, MAE: microwave-assisted extraction.

The interaction between temperature and ethanol concentration showed a significant effect (*p* < 0.05) on DPPH. High values of antioxidant activity were obtained at high temperature and ethanol concentration values (Figure 1). Under these conditions, the cell wall may be severely damaged, releasing an increased amount of antioxidant compounds. Furthermore, the diffusion coefficient increases with increasing temperature, facilitating the diffusion of different antioxidant compounds [12,61,62].

Some variability was found between MAE experimental conditions which optimized TPC and DPPH, individually. Consequently, a simultaneous multi-response optimization approach was carried out using the desirability function, resulting in optimum MAE conditions of 15 min, 80 °C, 63% ethanol and 80 mL (1/80 g mL^−1^ solid/liquid ratio), with a desirability value of 0.914. Predicted values obtained with the mathematical models were 1.52 ± 0.21 mg GAE g TS^−1^ and 1.28 ± 0.35 mg TE g TS^−1^, respectively. Verification experiments under optimal MAE conditions were performed, in triplicate, and the obtained responses for TPC and DPPH were 1.72 ± 0.04 mg GAE g TS^−1^ and 1.46 ± 0.02 mg TE g TS^−1^, respectively. These results confirmed the reliability of the proposed models to predict the studied responses.

### 3.2. Ultrasound-Assisted Extraction (UAE) Optimization

A BBD strategy was used to optimize UAE conditions with 17 runs which were performed randomly. The design matrix and results obtained for all experiments are shown in Table 2. The influence of three independent variables (amplitude, ethanol concentration and extraction time) in two responses (TPC and DPPH) was evaluated. All the studied responses were expressed as a function of independent variables by using second-order polynomial equations as follows (only significant factors):

TPC (mg GAE g TS^−1^) = −0.15485 + 0.03624A + 0.05614B + 0.00821C − 0.00031A^2^ + 0.00001AB − 0.00004AC − 0.00145B^2^ − 0.00018BC − 0.00002C^2^ (5)

DPPH (mg TE g TS^−1^) = –0.13093 + 0.04020A + 0.00021B + 0.00079C − 0.00032A^2^ + 0.00013AB + 0.00001AC − 0.00036B^2^ + 0.00006BC − 0.00001C^2^ (6)

where A, B and C represent ethanol concentration, extraction time and amplitude, respectively.

An ANOVA was carried out to evaluate the effect of the studied variables in the selected responses and to evaluate the reliability of the fitted models (Table 4). Adequate R^2^ values were obtained (0.9001 and 0.7858 for TPC and DPPH, respectively), with adjusted R^2^ values quite close to 1, confirming the accuracy of the fitted models in correlating predicted results with experimental data. Moreover, the high *p*-values showed for the lack of fit (0.1908 and 0.0590 for TPC and DPPH, respectively) indicated that it was not significant, confirming the good fitness of the models. Finally, the obtained CV values ranging from 4.98–8.07 % suggested high reproducibility of results and the reliability of the models to predict TPC and DPPH responses.

Ultrasonic waves applied in extraction processes are influenced by acoustic cavitation, which may cause cell walls disruption in the raw material matrix, promoting the release of antioxidant compounds. UAE is based on applying energy from ultrasonic waves, which causes compression and expansion cycles in the system. Due to the propagation of this mechanical vibration, acoustic cavitation happens, introducing pressure changes which cause the production, growth and collapse of a succession of microbubbles into the liquid phase. The implosion of bubbles occurs when the ultrasonic energy is not enough to maintain the vapor phase into the bubble. As a consequence, large amounts of energy are released which are responsible of sample tissue disruption [56,63,64].

The TPC values from TS by UAE ranged from 1.15 to 1.55 mg GAE g TS^−1^. The polyphenols extraction by UAE was mainly influenced by ethanol concentration and extraction time (*p* < 0.01). In contrast, the amplitude of ultrasound waves showed a positive effect, but it was not significant (*p* > 0.05), confirming that enough energy was supplied to the system to carry out the extraction process in this case. Ethanol concentration presented significant negative linear and square effects on TPC (*p* < 0.01), increasing the release of polyphenolic compounds by using a low ethanol concentration. The presence of water in the extraction solvent can act as a swelling agent of the TS matrix, increasing the contact surface, while ethanol can induce the breaking of the solute–matrix bond [65,66]. By increasing the contact surface, the probability of fragmentation attributed to particle collisions and ultrasonic waves also increases, causing a reduction in particle size and facilitating mass transfer [38]. Several authors reported the use of water–ethanol mixtures as an effective tool for polyphenol extractions as a consequence of a synergistic effect between both solvents [34,35,38]. Regarding extraction time, a significant positive linear effect (*p* < 0.01) was found, showing a direct correlation with extraction efficiency due to sample cells and solvent interaction in UAE [57]. The extraction temperature was kept almost constant (around 45 °C) during the experiments by using a refrigeration system to avoid the thermal degradation of extracted compounds. This procedure allowed increasing extraction time, and ultrasound waves exposure resulting in an increase in polyphenols extraction without showing significant degradation.

DPPH results were mainly influenced by ethanol concentration, showing a significant negative quadratic effect (*p* < 0.01), in agreement with TPC results, confirming that an intermediate water concentration can increase the extraction of compounds with high antioxidant activity. However, a significant positive linear effect (*p* < 0.01) was also found, suggesting that by using a high concentration of ethanol other type of compounds with antioxidant activity can be also extracted. El-Malah et al. reported similar antioxidant activity values using water and ethanol pure solvents by UAE from Egyptian tomato waste (composed of skin and seeds), which were higher compared to other extraction solvents, and they concluded that several solvents may have different selectivity for certain antioxidant compounds present in dry tomato waste [67]. Amplitude and extraction time were not significant (*p* > 0.05), although both factors showed a positive influence indicating that enough energy and extraction time were used to extract active compounds with high antioxidant activity. However, increasing their values, considering the studied surface area, did not provide a significant improvement in DPPH values.

Analogously to MAE optimization, a multi-response optimization procedure using the desirability function was carried out. The optimal UAE conditions found were 15 min, 61% ethanol and 85% amplitude (desirability = 0.952), using 1/50 g mL^−1^ solid/liquid ratio and 23.9 kHz. These conditions were validated by performing experimental analysis, in triplicate, obtaining 1.61 ± 0.03 mg GAE g TS^−1^ and 1.25 ± 0.01 mg TE g TS^−1^ for TPC and DPPH, respectively. These results were in close agreement with those found for the predicted values (1.53 ± 0.07 mg GAE g TS^−1^ and 1.29 ± 0.10 mg TE g TS^−1^ for TPC and DPPH, respectively), confirming the reliability of the proposed models to predict the studied responses.

### 3.3. Comparison between UAE and MAE Extracts

Different mechanisms are involved in the extraction process depending on the assisted technique. MAE is based on the interaction between microwaves and raw material while UAE is based on the cavitation phenomenon produced by ultrasound waves. Despite this, similar optimal conditions were found for extraction time and ethanol concentration with optimal values of 15 min in both techniques, and 63% and 61 % ethanol for MAE and UAE, respectively. In addition, the power values applied under optimal conditions for MAE and UAE were 92.7 ± 6.7 W and 103.1 ± 1.3 W, respectively; the result was that MAE was the process with the lowest energy consumption, since both processes were carried out during the same period time. The extracts (TSE) obtained at optimal MAE and UAE experimental conditions were fully characterized by using different analytical techniques in order to compare the efficiency of both extraction techniques.

#### 3.3.1. Morphological Characterization by SEM

**Figure 2:**
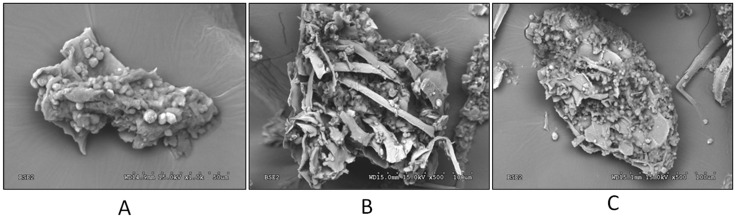
SEM micrographs of raw TS powder before extraction (**A**), and TS residue obtained after MAE (**B**) and UAE (**C**). TS: Tomato seed, UAE: ultrasound-assisted extraction.

The vegetal material damage produced after extraction in tomato seed samples was evaluated by SEM. Figure 2 shows micrographs obtained for raw tomato seeds after grinding (A) and tomato seed residue after MAE (B) and UAE (C) processes. A compact and rough initial appearance was observed for TS, before extraction, showing an irregular surface. In contrast, different morphological changes in the raw material surface after extraction were found. Microwave-treated TS sample showed an uneven surface which was associated with microwave-targeted strikes during MAE, presenting deep holes exposing the inner part of TS, probably due to the high dielectric constant of the solvent and its diffusion through the cell wall [66]. These changes suggested that MAE played an important role in breaking up TS cell walls, resulting in a greatly destroyed surface with a crumbled texture, due to the potential of electromagnetic waves to increase temperature and internal pressure inside the cells [49]. After UAE, TS presented large cavities on the surface as a consequence of solvent penetration, indicating severe damage on cell walls due to bubbles produced by acoustic cavitation, which aided in the disruption of TS cell walls [68]. In conclusion, compared to the raw initial material, the treatment of TS with MAE and UAE produced a critical deterioration of cells releasing polyphenols into the extraction solvent.

#### 3.3.2. Extraction Yield

Despite extraction yield was not considered to obtain optimal extraction conditions, values obtained under experimental tests performed in Table 1 and Table 3 ranged 7–10% and 8–12% for MAE and UAE, respectively. These results were similar to those reported by other authors from tomato processing by-products using conventional extraction techniques (8–14%) but involving longer extraction times (more than 60 min) compared to MAE or UAE. In addition, it has to be considered that the obtained yields depend on different variables, such as tomato variety, origin, maturity and ripening state [69,70]. Under optimal extraction conditions, extraction yields of 11.0 ± 0.1% and 13.2 ± 0.2% for MAE and UAE, respectively, were obtained, showing UAE had the highest extraction yield. These differences could be related to the presence of other compounds with high molecular weight that may have been released during the extraction process, due to an improvement in mass transfer and assistance of thermal treatment in UAE [67]. Chada et al. [71] found a yield of 6.42% when extracting antioxidant compounds from industrial tomato pomace by MAE using ethanol/ethyl acetate (90:10, *v/v*) as solvent, which was quite lower than the results obtained in the present work. Moreover, these authors reported a yield of 15.18% by pressurized liquid extraction (PLE) with ethanol/ethyl acetate (50:50, *v*/*v*), similar to yields obtained in our study by MAE and UAE. In contrast, Pinela et al. [58] reported an extraction yield of 35% in tomato by MAE using pure ethanol, which was related to the use of higher temperatures (180 °C) than those applied in this work. In a recent work, Panagiotopoulou et al. [72] reported yields around 35% and 50% for extracts obtained by UAE using an ultrasonic bath and ethanol–acetic acid (95:5, *v*/*v*) for 10 min in green and red tomato fruit wastes, respectively. These high yields were associated with a previous maceration pretreatment of samples, before extraction, for 72 h using the same solvent in order to soften the cell walls, overpassing the total extraction time used in this work. It should be taken into consideration that the aim of this study was the recovery of antioxidant compounds, and so, it is essential to focus the efficiency of the used extraction techniques in terms of total phenolic content (TPC) and antioxidant activity, as considering the overall yield will also include other compounds able to be co-extracted at optimal experimental conditions used both in MAE and UAE.

#### 3.3.3. FTIR

**Figure 3:**
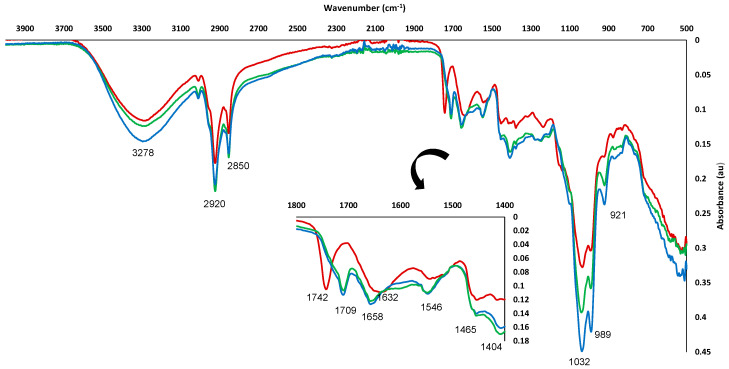
FTIR spectra of TS (−−−−−) and TSE obtained under optimum MAE (−−−−−) and UAE (−−−−−) conditions.

The FTIR spectra of the raw material (Figure 3) suggested the presence of different types of compounds in the TS sample. The strong peak appearing at 3278 cm^−1^ was associated with –OH stretching vibration of carboxylic groups. Aromatic and aliphatic groups were also present, being related to 2929 and 2934 cm^−1^ peaks. The region of 1800–1400 cm^−1^ gave special information on functional groups present in TS. The peak showed at 1742 cm^−1^, mainly assigned to C=O stretching vibration of alkyl ester, could be indicative of the presence of several polysaccharides, such as pectin, methyl esterified uronic acid, cellulose, heteromannans, heteroxylans and lignin. In addition, a broad band associated with COO- antisymmetric stretching C=O at 1632 cm^−1^ was assigned to free carbonyl groups, carboxylate, amide, and phenolic compounds. Finally, the peak appearing at 1546 cm^−1^ was related to lignin and phenolic ring/backbone due to the aromatic C=C vibration [73,74,75].

Similar FTIR spectra were obtained for MAE and UAE extracts (Figure 3). A wide variety of organic molecules with aromatic and phenolic groups were related to some typical characteristic peaks usually present in phenolic extracts, as reported by other authors [76,77]. A strong peak associated with O-H stretching of the phenol group in the range 3000–3600 cm^−1^ was observed. The peaks showed at 2923 and 2852 cm^−1^ were assigned to asymmetric and symmetric stretching vibrations of methylene groups (-CH_2_), respectively. Moreover, the peaks appearing at 1658, 1408, 1248 and 1038 cm^−1^ were related to C=C stretching vibration, OH stretching of alcohol or phenol groups, C-OH stretching and C-O stretching from methoxy groups of alcohols, ethers or esters, respectively. According to FTIR spectra, both MAE and UAE extracts exhibited similar main peaks, being mainly composed of similar groups of biomolecules, despite having been obtained through inherently different extraction methods.

#### 3.3.4. Total Phenolic Content and Antioxidant Activity

Table 5 shows the results obtained for TPC and antioxidant activity using different spectrophotometric assays, under MAE and UAE optimal extraction conditions. Significant differences (*p* < 0.05) were found regarding TPC values (1.72 ± 0.04 and 1.61 ± 0.03 mg GAE g TS^−1^ for MAE and UAE, respectively), showing higher results for MAE extracts. Lasunon et al. [78] obtained a TPC value of 280.10 mg GAE 100 g^−1^ from industrial tomato waste by MAE using 95% ethanol, which agrees with the result obtained in this work considering that samples studied by these authors were composed of seed, pulp, and skin, in which bioactive compounds present in each part may be different. Abbassi et al. [79] reported a TPC value of 18.74 mg GAE 100 g^−1^ in tomato seed extracts obtained by Soxhlet extraction with ethanol, which was much lower than the values obtained in our work by using MAE and UAE. In another study, El-Malah et al. reported a TPC value of 162.50 mg GAE 100 g^−1^ from Egyptian tomato waste (skin and seeds) by UAE using ethanol, which is very close to the result found in our work [67]. Finally, similar TPC values were reported by other authors from tomato by-products (peels and seeds) using ohmic heating extraction [22,66], confirming the great efficiency of MAE and UAE techniques to obtain extracts rich in polyphenols.

Considering that different extraction mechanisms are involved in MAE and UAE, i.e., application of microwaves or ultrasounds, it could be expected that different bioactive compounds may also be extracted from TS, with these techniques having their own effect on extracting individual phenolic compounds. UAE is based on cavitation phenomena, and so, compounds located closer to the surface of the tomato cell membrane can be more easily extracted [80]. Nayak et al. [81] obtained higher TPC values by MAE compared to UAE for the recovery of polyphenols from *Citrus sinensis* peels, and this effect was attributed to the ability of the microwaves to penetrate the cell matrix and interact with polar molecules, resulting in the volumetric heating of the material, increasing pressure inside the plant cell, resulting in breaking of cell walls and the release of phenolic compounds.

Three different fast and reproducible methods were used to evaluate the antioxidant activity of TSE: DPPH and ABTS radical scavenging assays and FRAP as an *electron*-transfer reaction-based assay. These tests are reported as the most common used to analyze the antioxidant activity of natural extracts [82]. Significant (*p* < 0.05) differences between TSE obtained by MAE and UAE were found, showing better antioxidant results for MAE extracts in all assays (Table 5), suggesting the potential of this technique to isolate active compounds with antioxidant properties. This higher antioxidant activity of MAE extract could be explained considering that the microwave treatment may affect the structure of the cell due to the sudden increase in temperature and internal pressure, promoting the destruction of sample surface in agreement with the results obtained by SEM, as a result of the direct effect of microwaves on molecules by ionic conduction and dipole rotation. So, although ultrasound waves can break the cell wall, due to the cavitation phenomenon, and release phenolic compounds into the extraction solvent, the quantity of the extracted analytes will depend on the intensity and duration of the ultrasounds application [81].

MAE has been successfully used to obtain polyphenols from different agro-food wastes such as apple peels [83] or avocado seeds [84]. The antioxidant activity of the obtained compounds depends on polyphenols type, which can be related to differences found in MAE and UAE extracts [9,22,24,85]. In addition, the co-extraction of other compounds present in high concentrations in TS, such as lycopene or β-carotene, which also have high antioxidant capacity, have to be considered [86,87]. Antioxidant activity also depends on the harvesting period as a consequence of changes in the phenolic profile, as different individual polyphenols will be developed and/or accumulated during the plant growth [88,89]. In conclusion, the differences found between TPC and antioxidant capacity values in MAE and UAE extracts may be related to both quantity and type of phenolics present in these extracts.

#### 3.3.5. Main Phenolics Analyzed by HPLC-DAD-MS

Three main phenolic compounds present in TSE were identified and quantified by HPLC-DAD-MS (Table 6). Acceptable levels of linearity were obtained for all calculated calibration curves with R^2^ values ranging between 0.9984 and 0.9993 for the studied analytes, at six calibration points run in triplicate (Table 6). Repeatability was evaluated by analyzing standard solutions, in triplicate, within the same day with relative standard deviation (RSD) values ranging between 2.5% and 6.9%. LOD and LOQ values obtained ranged 0.04–0.18 mg kg^−1^ and 0.15–0.61 mg kg^−1^, respectively.

The major phenolic compounds present in TSE obtained by MAE and UAE were chlorogenic acid, rutin and naringenin. These results were in close agreement with those reported by other authors in different tomato seed samples. Grassino et al. [7] reported chlorogenic acid and its derivates as the major compounds present in tomato peel waste (86.13  ±  0.48 mg 100 g^−1^) by using UAE. Other compounds such as caffeic acid or quercetin derivates were also found. In another work, high-hydrostatic pressure extraction (HHPE) was used for extracting polyphenols from tomato peel waste generated by the canning industry, and the authors found *p*-coumaric acid and chlorogenic acid derivative as predominant compounds with contents of 0.57 to 67.41 mg kg^−1^ and 1.29 to 58.57 mg kg^−1^, respectively [42], which are lower than results obtained by MAE and UAE in our study, thus highlighting the impact of the extraction technique used in recovering polyphenols from tomato wastes. Rutin and naringenin flavonoids were also identified in whole tomato samples with a concentration ranging 2.55–43.31 mg kg^−1^ and 0.46–4.74 mg kg^−1^, respectively, together with chlorogenic acid ranging from 16.81–99.65 mg kg^−1^, depending on tomato variety by using a conventional extraction method with methanol–water mixtures [90].

Significantly (*p* < 0.05) higher concentrations of all analyzed compounds were obtained for MAE extracts, in agreement with results found for TPC and antioxidant activity, showing MAE as the most effective extraction method to obtain polyphenolic compounds with antioxidant activity from TS under the studied conditions. These results also suggested that MAE extracts could have higher purity and antioxidant capacity compared to those obtained by UAE, as a consequence of the different extraction mechanisms explained in previous sections.

#### 3.3.6. Thermal Properties

**Figure 4:**
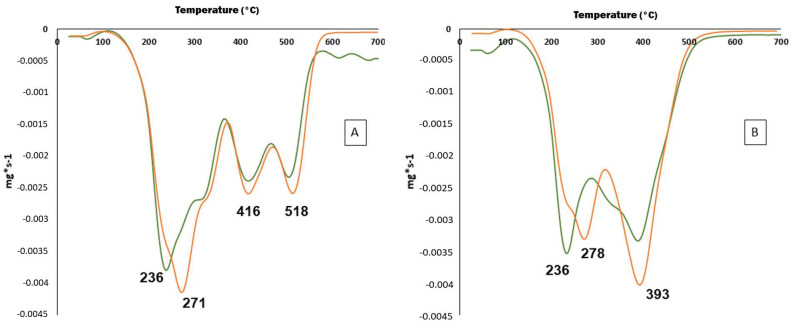
Thermograms of TSE obtained under optimum MAE (**--------**) and UAE (**------**) conditions in nitrogen (**A**) and oxygen (**B**) atmospheres.

The DTGA curve of TSE samples showed three main degradation steps in both inert and oxygen atmospheres (Figure 4). The thermal degradation process in both cases occurred over a wide range of temperatures, showing multiple overlapped decomposition steps, in agreement with the different compounds present in TSE. The first degradation step found in inert atmosphere showed Tmax values of 236 ± 1 °C and 271 ± 2 °C for MAE and UAE, respectively (Figure 4A). These results suggested the presence of some compounds with high molecular weight in TSE obtained by UAE, as it is well known that increasing molecular weight results in an increase in Tmax values [91]. Similar Tmax values were obtained in oxygen atmosphere (Figure 4B) confirming the good thermal stability of TSE. However, a reduction in Tmax values was found in the last degradation step using oxygen atmosphere as a consequence of the oxidation of the compounds present in both TSE samples [92].

Similar TGA results were reported in natural extracts from *Aloe vera* leaves [93] or Yerba mate [94]. According to Figure 4, the first degradation step could be associated with the degradation of some low molecular weight components of TSE, such as those described in Table 6. Finally, the third and fourth thermal degradation steps were related to high molecular weight compounds such as cellulose and lignin derivatives present in TSE. According to the obtained results, optimal experimental conditions used for MAE and UAE should not produce significant thermal degradation of active compounds present in TS. The excellent thermal properties found for both TSE obtained by MAE and UAE techniques have shown potential applications for their incorporation in different polymers to obtain functional materials using high processing temperatures.

## 4. Conclusions

In this study, microwave-assisted extraction (MAE) and ultrasound-assisted extraction of tomato seed industrial wastes were optimized by using Box–Behnken experimental designs, for the first time. A high reliability of the developed models was obtained with optimal MAE and UAE conditions of 15 min, 80 °C, 63% ethanol, 80 mL (desirability = 0.914) and 15 min, 61% ethanol, 85% amplitude (desirability = 0.952), respectively. Extracts rich in polyphenols with high antioxidant activity were obtained by MAE and UAE, with MAE showing higher results in terms of TPC, DPPH, ABTS and FRAP values. MAE extracts also exhibited higher individual contents of chlorogenic acid, rutin and naringenin, which were quantified by HPLC-DAD-MS. This behavior was related to the microwave volumetric and selective heating mechanism, promoting the destruction of sample surface as a consequence of the sudden increase in temperature and internal pressure produced, releasing a higher content of phenolic compounds. In contrast, UAE is more influenced by the intensity and duration of the ultrasound application, and so, the quantity of extracted phenolics from the food sample. This explanation is in line with results obtained by SEM, where a critical deterioration of cells was observed by MAE, expecting a higher release of polyphenols into the extraction solvent. Good thermal properties were found for MAE and UAE extracts with maximum degradation temperatures starting above 200 °C in both inert and oxidative atmospheres, allowing their incorporation in materials through processes involving high temperatures. In summary, MAE and UAE extracts obtained from tomato seed wastes have shown antioxidant potential applications to be applied in different sectors such as food, nutraceuticals, and cosmetics industries. This research can provide useful information for tomato industrial processors and can also be a basis for further scaling and industrial transferring of the developed green extraction processes, also contributing to the circular economy approach by valorizing the studied wastes.

## Figures and Tables

**Table 1:** Box–Behnken experimental design matrix and response values obtained from TS by MAE.

	Experimental Design				Responses	
Run	t (min)	T (°C)	EtOH (%)	V (mL)	TPC (mg GAE g TS^−1^)	DPPH (mg TE g TS^−1^)
1	15	60	80	65	1.30 ± 0.03	1.17 ± 0.01
2	15	40	60	65	1.35 ± 0.01	1.12 ± 0.04
3	15	80	60	65	1.50 ± 0.04	1.20 ± 0.01
4	10	60	60	65	1.43 ± 0.04	1.14 ± 0.03
5	10	60	80	80	1.28 ± 0.03	1.12 ± 0.03
6	10	60	40	50	1.52 ± 0.01	1.04 ± 0.14
7	10	60	60	65	1.47 ± 0.01	1.14 ± 0.04
8	10	40	60	80	1.27 ± 0.03	1.05 ± 0.01
9	10	60	40	80	1.49 ± 0.02	0.95 ± 0.04
10	15	60	60	50	1.39 ± 0.04	1.09 ± 0.03
11	10	40	80	65	1.09 ± 0.02	0.96 ± 0.03
12	10	80	60	50	1.52 ± 0.05	1.16 ± 0.04
13	15	60	40	65	1.55 ± 0.04	1.00 ± 0.04
14	5	60	60	80	1.46 ± 0.02	1.13 ± 0.05
15	10	80	60	80	1.59 ± 0.03	1.26 ± 0.01
16	5	60	80	65	1.25 ± 0.01	1.11 ± 0.03
17	10	60	60	65	1.44 ± 0.03	1.16 ± 0.01
18	10	60	60	65	1.41 ± 0.03	1.14 ± 0.01
19	10	80	80	65	1.39 ± 0.02	1.23 ± 0.06
20	10	40	60	50	1.28 ± 0.04	1.05 ± 0.01
21	5	60	60	50	1.41 ± 0.03	1.14 ± 0.01
22	10	40	40	65	1.34 ± 0.06	0.97 ± 0.08
23	15	60	60	80	1.52 ± 0.02	1.22 ± 0.02
24	5	60	40	65	1.52 ± 0.04	0.96 ± 0.02
25	10	80	40	65	1.62 ± 0.03	1.06 ± 0.03
26	10	60	60	65	1.47 ± 0.02	1.20 ± 0.01
27	5	40	60	65	1.27 ± 0.01	1.05 ± 0.01
28	5	80	60	65	1.53 ± 0.03	1.24 ± 0.01
29	10	60	80	50	1.29 ± 0.02	1.18 ± 0.13

GAE: Gallic acid equivalents. TE: Trolox equivalents.

**Table 2:** Box–Behnken experimental design matrix and response values obtained from TS by UAE.

	Experimental Design			Responses	
Run	EtOH (%)	t (min)	A (%)	TPC (mg GAE g TS^−1^)	DPPH (mg TE g TS^−1^)
1	60	10	70	1.44 ± 0.02	1.19 ± 0.02
2	60	10	70	1.53 ± 0.03	1.23 ± 0.01
3	60	10	70	1.55 ± 0.01	1.27 ± 0.01
4	80	15	70	1.37 ± 0.02	1.17 ± 0.01
5	60	10	70	1.49 ± 0.01	1.30 ± 0.01
6	40	10	40	1.36 ± 0.00	0.99 ± 0.01
7	40	15	70	1.54 ± 0.01	1.11 ± 0.01
8	80	10	100	1.31 ± 0.02	1.25 ± 0.01
9	80	5	70	1.15 ± 0.01	1.10 ± 0.01
10	60	15	40	1.48 ± 0.03	1.23 ± 0.01
11	80	10	40	1.31 ± 0.01	1.24 ± 0.00
12	60	10	70	1.51 ± 0.03	1.28 ± 0.01
13	60	5	100	1.46 ± 0.02	1.23 ± 0.03
14	40	10	100	1.45 ± 0.01	0.99 ± 0.01
15	60	5	40	1.35 ± 0.01	1.19 ± 0.01
16	40	5	70	1.32 ± 0.01	1.09 ± 0.00
17	60	15	100	1.49 ± 0.02	1.30 ± 0.01

TPC: Total Phenolic Content. GAE: Gallic acid equivalents. TE: Trolox equivalents.

**Table 3:** ANOVA results for response surface quadratic models of TS extraction by MAE.

Source	Sum of Squares	Df	Mean Square	F-Ratio	*p*-Value
**TPC**					
A	0.0024	1	0.0024	3.54	0.1330
B	0.2002	1	0.2002	294.42	0.0001 ***
C	0.1728	1	0.1728	254.12	0.0001 ***
D	0.0033	1	0.0033	4.90	0.0912
AA	0.0001	1	0.0001	0.14	0.7271
AB	0.0030	1	0.0030	4.45	0.1026
AC	0.0001	1	0.0001	0.15	0.7209
AD	0.0016	1	0.0016	2.35	0.1998
BB	0.0074	1	0.0074	10.81	0.0303 *
BC	0.0001	1	0.0001	0.15	0.7209
BD	0.0016	1	0.0016	2.35	0.1998
CC	0.0146	1	0.0146	21.45	0.0098 **
CD	0.0001	1	0.0001	0.15	0.7209
DD	4.5045 × 10 ^−8^	1	4.5045 × 10 ^−8^	0.00	0.9939
Lack-of-fit	0.0116	10	0.0012	1.71	0.3180
Pure error	0.0027	4	0.0007		
Total (corr.)	0.4211	28			
R^2^	0.9659				
Adj R^2^	0.9317				
CV (%)	4.89				
**DPPH**					
A	0.0024	1	0.0024	3.54	0.1330
B	0.0752	1	0.0752	110.60	0.0005 ***
C	0.0520	1	0.0520	76.48	0.0009 ***
D	0.0004	1	0.0004	0.60	0.4817
AA	0.0001	1	0.0001	0.03	0.8726
AB	0.0030	1	0.0030	4.45	0.1026
AC	0.0001	1	0.0001	0.15	0.7209
AD	0.0049	1	0.0049	7.21	0.0550
BB	0.0009	1	0.0009	1.32	0.3151
BC	0.0081	1	0.0081	11.91	0.0260 *
BD	0.0025	1	0.0025	3.68	0.1277
CC	0.0488	1	0.0488	71.79	0.0011 **
CD	0.0002	1	0.0002	0.33	0.5959
DD	0.0003	1	0.0003	0.43	0.5458
Lack-of-fit	0.0184	10	0.0018	2.70	0.1754
Pure error	0.0027	4	0.0007		
Total (corr.)	0.2206	28			
R^2^	0.9044				
Adj R^2^	0.8088				
CV (%)	7.25				

A: extraction time; B: extraction temperature; C: ethanol concentration; D: solvent volume. * significant effect at *p* < 0.05, ** significant effect at *p* < 0.01, *** significant effect at *p* < 0.001.

**Table 4:** ANOVA results for response surface quadratic models of TS extraction by UAE.

Source	Sum of Squares	Df	Mean Square	F-Ratio	*p*-Value
**TPC**					
A	0.0370	1	0.0370	23.37	0.0084 **
B	0.0450	1	0.0450	28.41	0.0060 **
C	0.0048	1	0.0048	3.07	0.1544
AA	0.0642	1	0.0642	40.52	0.0031 **
AB	0.0000	1	0.0000	0.00	0.9721
AC	0.0021	1	0.0021	1.33	0.3127
BB	0.0055	1	0.0055	3.50	0.1349
BC	0.0029	1	0.0029	1.83	0.2471
CC	0.0019	1	0.0019	1.19	0.3364
Lack-of-fit	0.0123	3	0.0041	2.58	0.1908
Pure error	0.0063	4	0.0016		
Total (corr.)	0.1862	16			
R^2^	0.9001				
Adj R^2^	0.7715				
CV %	4.98				
**DPPH**					
A	0.041	1	0.041	21.75	0.0096 **
B	0.0049	1	0.0049	2.60	0.1823
C	0.0024	1	0.0024	1.27	0.3220
AA	0.0690	1	0.0690	36.77	0.0037 **
AB	0.0006	1	0.0006	0.34	0.5900
AC	0.0001	1	0.0001	0.04	0.8468
BB	0.0003	1	0.0003	0.18	0.6958
BC	0.0003	1	0.0003	0.17	0.6977
CC	0.0003	1	0.0003	0.15	0.7192
Lack-of-fit	0.0253	3	0.0084	4.49	0.0905
Pure error	0.0075	4	0.0019		
Total (corr.)	0.1531	16			
R^2^	0.7858				
Adj R^2^	0.5103				
CV %	8.07				

A: ethanol concentration; B: extraction time; C: amplitude. ** significant effect at *p* < 0.01.

**Table 5:** Characterization of TSE obtained under optimum MAE and UAE conditions (*n* = 3; mean ± SD). Different superscripts within the same row indicate significant differences between extracts (*p* < 0.05).

Response	MAE	UAE
TPC (mg GAE g TS^−1^)	1.72 ± 0.04 ^A^	1.61 ± 0.03 ^B^
DPPH (mg TE g TS^−1^)	1.46 ± 0.03 ^A^	1.25 ± 0.01 ^B^
FRAP (mg TE g TS^−1^)	2.29 ± 0.04 ^A^	1.86 ± 0.01 ^B^
ABTS (mg TE g TS^−1^)	2.71 ± 0.02 ^A^	2.23 ± 0.01 ^B^

**Table 6:** Main polyphenols quantified in TSE under optimum MAE and UAE conditions (*n* = 3; mean ± SD). Different superscripts within the same row indicate significant differences between extracts (*p* < 0.05).

Compound	[M-H]- (*m*/*z*)	RT (min)	R^2^	LOD (mg kg^−1^)	LOQ (mg kg^−1^)	RSD (%)	MAE (mg 100 g TS^−1^)	UAE (mg 100 g TS^−1^)
Chlorogenic acid	353	3.6	0.9984	0.18	0.61	2.9	1.11 ± 0.35 ^A^	0.58 ± 0.06 ^B^
Rutin	609	7.7	0.9993	0.09	0.29	6.2	1.38 ± 0.02 ^A^	0.75 ± 0.09 ^B^
Naringenin	271	17.1	0.9992	0.04	0.15	2.5	2.99 ± 0.11 ^A^	1.93 ± 0.07 ^B^

Sy/x = standard deviation of residues, m = slope. LOD: limit of detection. Calculated for 3 S_y/x_/m. LOQ: limit of quantification. Calculated for 10 S_y/x_/m.

## Data Availability

Data are contained within the article.

## Abbreviations

%EtOH, ethanol concentration; A, amplitude; ABTS, 2,2’-azino-bis(3-ethylbenzothiazoline-6-sulfonic acid) diammonium salt; ANOVA, analysis of variance; ATR, attenuated total reflectance; BBD, Box–Behnken design; CV, coefficient of variation; DAD, diode array detector; DPPH, 2,2-diphenyl-1-picrylhydrazyl; FAO, Food and Agriculture Organization; FRAP, ferric-reducing antioxidant power; FTIR, Fourier-transform infrared spectroscopy; GAE, gallic acid equivalents; HHPE, high-hydrostatic pressure extraction; HPLC, high-performance liquid chromatography; LOD, limit of detection; LOQ, limit of quantification; m, slope; MAE, microwave-assisted extraction; MS, mass spectrometry; PLE, pressurized liquid extraction; R^2^, coefficient of determination; RSD, relative standard deviation; RSM, response surface methodology; RT, retention time; SD, standard deviation; SEM, scanning electron microscopy; SSICA, Stazione Sperimentale per l’Industria delle Conserve Alimentari; Sy/x, standard deviation of residues; T, extraction temperature; t, extraction time; TE, Trolox equivalents; TGA, thermogravimetric analysis; Tmax, temperature of maximum degradation rate; TPC, total phenolic content; TPTZ, 2,4,6-tris(2-pyridyl)-s-triazine; TS, tomato seed; TSE, tomato seed extract; UAE, ultrasound-assisted extraction; V, solvent volume.
